# A protein-based subunit vaccine with biological adjuvants provides effective protection against *Pasteurella multocida* in pigs

**DOI:** 10.1186/s13567-023-01150-4

**Published:** 2023-03-02

**Authors:** Min-Chia Wu, Hsing-Chieh Wu, Jai-Wei Lee, Wan-Chen Chang, Chun-Yen Chu

**Affiliations:** 1 International Degree Program in Animal Vaccine Technology, International College, National Pingtung University of Science and Technology, Pingtung, 91201 Taiwan; 2 Graduate Institute of Animal Vaccine Technology, College of Veterinary Medicine, National Pingtung University of Science and Technology, Pingtung, 91201 Taiwan; 3 Department of Tropical Agriculture and International Cooperation, National Pingtung University of Science and Technology, Pingtung, Taiwan

**Keywords:** biological adjuvant, *Pasteurella multocida* toxin, suilysin, recombinant protein, subunit vaccine

## Abstract

*Streptococcus suis* (*S. suis*) and *Pasteurella multocida* (*P. multocida*) are pathogens that can cause zoonotic diseases. *P*. *multocida* toxin (PMT) is an important virulence factor that causes atrophic rhinitis in pigs. Suilysin (Sly) is an extracellular protein of *S. suis* and has been shown to be a potential adjuvant. Previous studies have indicated that subunit vaccines containing several fragments of PMT as antigens are safer than traditional inactivated or live-attenuated vaccines. However, protein-based vaccines need strong adjuvants to enhance their immunogenicity. In this study, recombinant PMT-NC (rPMT-NC) protein antigen was formulated with either recombinant Sly (rSly) or CpG oligodeoxynucleotides (CpG) as the adjuvant. The immune responses elicited by these vaccines and the protective efficacy after challenge with live *P. multocida* were evaluated in piglets. In the dose-dependent test, piglets immunized with the low dose (100 µg) of rSly had increased antigen-specific total IgG, interferon (IFN)-γ gene expression, and CD4^+^ and CD8^+^ T-cell populations. Compared to piglets in the commercial (Al-gel) adjuvant and the control groups (*p* < 0.05), piglets in the biological adjuvant groups showed significantly reduced turbinate atrophy, nasal distortion, and lung lesion scores after challenge with *P. multocida* serotype A. Vaccines containing rSly or CpG adjuvant enhanced humoral and cellular immune responses and protection against *P. multocida*. This combination of a protein-based antigen formulated with a biological adjuvant showed synergistic and protective effects against atrophic rhinitis and has potential to be developed as part of a bivalent vaccine.

## Introduction

Porcine respiratory disease complex (PRDC) is caused by coinfection with many pathogens, including porcine circovirus 2 (PCV 2), porcine reproductive and respiratory virus (PRRSV), *Mycoplasma hyopneumoniae*, *Actinobacillus pleuropneumoniae*, *Pasteurella multocida* (*P. multocida*) and *Streptococcus suis* (*S. suis*) [1]. *P. multocida* can cause pneumonia and porcine atrophic rhinitis (AR), leading to facial distortion, turbinate bone destruction and reduced growth of piglets [2]. *S. suis* is an important zoonotic pathogen that causes arthritis, meningitis, septicemia and sudden death in pigs [3]. Pigs infected with a single pathogen have mild symptoms, but mixed infections can cause severe symptoms and even death [4]. Vaccination is the best strategy to prevent these diseases. The success of traditional vaccines (inactivated, live-attenuated) has decreased livestock industry economic losses. However, inactivated vaccines lack cross-protection, and live-attenuated vaccines risk reverting to virulence [5]. Thus, a safe and efficient vaccine to prevent these diseases is essential.

In recent years, many subunit vaccine formulations have been developed. However, most subunit antigens are poorly immunogenic and require adjuvants to help enhance the adaptive immune response and effector T-cell functions [6]. Adjuvants are important ingredients of vaccines that stimulate immune responses to facilitate antigen recognition in both humans and animals [7]. Many diverse compounds have been licenced as adjuvants, including mineral salts (aluminium salts), emulsions (MF59 or ISA 206), liposomes, microbial derivatives (CpG or rSly) and cytokines (dendritic cells or IL-12) [8]. However, the strategies used to develop adjuvants may vary and depend on the type of vaccine.

Several biological adjuvants are pathogen-associated molecular patterns (PAMPs), including suilysin (Sly) and unmethylated CpG dinucleotide motifs (CpG ODNs) [9]. PAMPs are repetitive molecules that are present in microorganisms [10] and are designed for specific pattern recognition receptors (PRRs), such as Toll-like receptors (TLRs). As a result, they activate dendritic cells and macrophages and trigger key cytokines such as IL-4 or IL-12. The specific microbial product may enhance either the Th1 or Th2 responses. Recently, by using well-understood PAMPs, the formulation and development of vaccines have led to safer and more effective stimulation of immune responses by reducing the side effects of other adjuvants.

Sly is a virulence factor of *S. suis* that triggers cytotoxic T-cell and natural killer cell maturation and activates the Th1 and Th2 immune responses [11]. CpG ODN is recognized by TLR 9, which triggers Th1 proinflammatory cytokines, as well as both innate and adaptive immune responses [12]. Our patented plasmid CpG ODN has been shown to be a candidate adjuvant in duck vaccines [13]. CpG ODN effectively enhances the immune responses conferred by adjuvants and is worth further investigation in swine vaccines.

*Pasteurella multocida* toxin (PMT) has been shown to be a virulence factor causing porcine atrophic rhinitis [2]. The N-terminal domain of PMT serves as a cell-binding domain, and the C-terminal domain acts as a catalytic region [14]. We previously designed a vaccine containing recombinant PMT (rPMT) engineered to have both the N- and C-termini of PMT (rPMT-NC) and formulated with recombinant Sly (rSly) or other conventional adjuvants (aluminium hydroxide, water-in-oil-in-water) [11]. Mice immunized with rPMT-NC + rSly had augmented antibody production and cellular immunity (increased cytokine expression and lymphocyte proliferation) compared to mice immunized with rPMT-NC plus other adjuvants. In addition, the survival rate of mice immunized with rPMT-NC + rSly was the highest (70%) compared to that of mice immunized with rPMT-NC alone (30%) after challenge with live *P. multocida* bacteria. However, commercially available vaccines contain killed *P. multocida* and three segments of rPMT formulated with Al-gel as the adjuvant. Developing new antigens and adjuvants is required to enhance the protection efficacy of vaccines against *P. multocida.*

This study aims to elucidate the synergistic effects of biological and traditional adjuvants using a simple protein-based antigen. The expressed rPMT-NC protein was combined with rSly or patented CpG adjuvant to evaluate protective immune responses in piglets challenged with *P. multocida*.

## Materials and methods

### *P*.* multocida* serotype A culture conditions

*Pasteurella multocida* was isolated from infected piglets in Taiwan. Primers for multiplex PCR to detect *P. multocida* serotype A were designed in our previous study [15]. *P. multocida* was cultivated in BHI (Brain Heart Infusion, BD, MD, USA) medium containing 3% chicken serum. Shaking for 6 h culture at 37 °C was used for challenge.

### Antigen and adjuvant preparation

The cloning and construction of the expression vector for rPMT-NC and rSly were performed as described previously [11]. *Escherichia coli* strain BL21 (DE3) (Invitrogen, CA, USA) harbouring the *PMT-NC* and *Sl*y recombinant plasmids were cultured in LB broth (Luria–Bertani, Difco, MD, USA) medium and incubated at 37 °C overnight, and the procedures for purification were as previously described.

The construction of a CpG plasmid containing 12 copies of the GACGTT motif is described in the Taiwan patent entitled “DNA adjuvant for waterfowl and live-stock vaccines” (Patent No. I425091), which is available for viewing online through the Intellectual Property Office, Taiwan. Plasmids were amplified in *E. coli*, purified using a QIAGEN-tip 500 (Qiagen, Hilden, Germany) and dissolved in sterile PBS for formulation. The purified rPMT-NC (200 μg/mL) as an antigen was emulsified with 50% water-in-oil-in-water (w/o/w) adjuvant (ISA206, Seppic, France), CpG plasmid (200 μg/mL) or rSly protein (100 μg/mL) and stored at 4 °C. The sterility of the vaccines was confirmed by culture with trypticase soy agar (Difco, MD, USA) (TSA, 37 °C), thioglycollate agar (Difco) (TGC, 37 °C) and Sabouraud dextrose agar (Difco) (SDA, 25 °C). All vaccine dosages had a final volume of 2 mL.

### Animal experiments in piglets

The Landrace–Yorkshire–Duroc (LYD) cross-bred piglets used in this study were bred and housed in an aseptic environment to protect them from opportunistic infections. All efforts were made to minimize animal suffering, and the animals were humanely sacrificed at the end of the study. All experimental protocols for the animal trials were approved (NPUST-103-014) by the Animal Care and Uses Committee at the National Pingtung University of Science and Technology. The experiments were conducted according to the Ethical Rules and Laws of NPUST.

### Vaccination and challenge of piglets

#### Animal trial 1-plasmid CpG adjuvant effect test

Twelve healthy 4-week-old piglets without *P. multocida* antibodies were randomly divided into three groups (*n* = 4 each). The vaccinated groups were immunized intramuscularly with 2 mL of (1) rPMT-NC + CpG, (2) rPMT-NC + w/o/w, or (3) PBS (Table 1). Piglets were boosted with the same vaccine at 2 weeks after the primary immunization. All piglets were challenged intranasally with 1 × 10^8^ CFU/mL *P. multocida* serotype A 4 weeks after primary immunization [16]. The antibody titre was detected by blood samples taken at 0, 2 and 4 weeks after primary immunization. The piglets were monitored daily for clinical signs, body temperature (fever was defined as rectal temperature > 39.5 °C), and body weight and were sacrificed for necropsy 14 days after challenge. Pathological examination was performed by the Veterinary Pathology Department of NPUST, and the lesion score was calculated based on the area of lesions in an organ, where no lesion = 0, lesion area < 33% = 1, lesion area 33–66% = 2, and lesion area > 66% = 3 [3].

**Table 1:** **The active ingredients of vaccines in this study**

Vaccine^a^	Adjuvant
Trial 1. Plasmid CpG adjuvant effect test	
1. rPMT-NC^b^ + CpG	CpG (200 μg/mL)
2. rPMT-NC + w/o/w	w/o/w
3. Control (PBS)	–
Trial 2. rSly adjuvant dose-dependent test	
4. rPMT-NC + w/o/w + rSly	rSly (100 μg/mL)
5. rPMT-NC + w/o/w + rSly	rSly (150 μg/mL)
6. Control (PBS)	–
Trial 3. Comparison with various adjuvants	
7. rPMT-NC + w/o/w + rSly	rSly (100 μg/mL)
8. rPMT-NC + w/o/w + CpG	CpG (200 μg/mL)
9. rPMT-NC	–
10. Commercial vaccine^c^	Al-gel
11. Control (PBS)	–

^a^Pig immunizaction vaccine with 2 mL by I.M.

^b^rPMT-NC (200 μg/mL).

^c^Ingredient: *B. bronchiseptica* (1 × 10^9^ CFU), *P. multocida* type A (1 × 10^9^ CFU).

*P. multocida* type D (1 × 10^9^ CFU), rsPMT/tox1 (20 μg), rsPMT/tox2 (20 μg), rsPMT/tox7 (20 μg), Adjuvant: Al-gel.

#### Animal trial 2-rSly adjuvant dose-dependent test

Twelve healthy piglets without *P. multocida* antibodies were divided into three groups (*n* = 4 each). The vaccinated groups were immunized intramuscularly with 2 mL of (4) rPMT-NC + w/o/w + rSly (100 μg/mL), (5) rPMT-NC + w/o/w + rSly (150 μg/mL), or (6) PBS as a negative control (Table 1). The antibody titre was determined for blood samples taken at 0, 2 and 4 weeks after primary immunization. CD4^+^ and CD8^+^ T-cell analysis was performed at week 4.

#### Animal trial 3-comparison with various adjuvants and commercial vaccines

Twenty healthy piglets without *P. multocida* antibodies were divided into five groups (*n* = 4 each). The vaccinated groups were immunized intramuscularly with 2 mL of (7) rPMT-NC + w/o/w + rSly, (8) rPMT-NC + w/o/w + CpG, (9) rPMT-NC without adjuvant, (10) commercial vaccine (*B. bronchiseptica*, *P. multocida* type A and D, rsPMT/tox 1, tox 2 and tox 7, Al-gel), or (11) PBS as a negative control (Table 1). Piglets were boosted with the same vaccine 2 weeks after primary immunization. All piglets were challenged intranasally with 1 × 10^8^ CFU/mL *P. multocida* serotype A 4 weeks after primary immunization. The antibody titre was detected in blood samples taken at 0, 2 and 4 weeks after primary immunization. The piglets were monitored daily for clinical signs and body weight and then sacrificed for necropsy 14 days after challenge. Pathological examination was performed as in trial 1.

### Determination of antibody titres with indirect ELISA

Flat-bottomed, polystyrene 96-well ELISA plates (ExtraGENE, Davis, CA) were coated with *P. multocida* (2.5 μg/mL, inactivated with 0.5% formalin) [11] in the coating buffer (15 mM Na_2_CO_3_, 35 mM NaHCO_3_, 3 mM NaN_3_, pH 9.6) at 4 °C overnight. The plates were washed with PBS containing 0.5% Tween-20 (PBS-T) three times and blocked with 1% BSA (KPL, Gaithersburg, MD, USA) in PBST at 37 °C for 1 h. Diluted pig serum (1:500) was added into the wells (50 μL/well) and incubated at 37 °C for 1.5 h. Thereafter, the plates were washed 5 times with PBST, and horseradish peroxidase (HRP)-conjugated goat anti-swine IgG-HRP (1:10 000) (KPL, Gathersburg, MD) was added (100 μL/well). The plates were incubated at 37 °C for 1 h and washed 7 times with PBST, and 100 μL TMB 2-Component Microwell Peroxidase substrate (KPL) was added to each well. After 5 min, the reaction was stopped by adding 100 μL of TMB stop solution (KPL), and the absorbance was measured at 450 nm using a multiwell plate reader (Anthos 2020; Anthos, Cambridge, UK). Samples were evaluated by examining the sample to positive ratio (S/P ratio) calculated as (Sample OD value − Negative OD value)/(Positive OD value − Negative OD value).

### Isolation of peripheral blood mononuclear cells (PBMCs)

PBMCs were isolated from blood samples collected at 0 and 4 weeks after primary immunization. Whole blood was mixed with Ficoll-Paque (GE Healthcare, St. Giles, Sweden), and the mixture was centrifuged at 525 × *g* for 30 min. The fraction containing PBMCs was collected, and the cells were washed twice and resuspended in RPMI-1640 (1 × 10^6^ cells/mL).

### Immunofluorescence analysis of CD4^+^ and CD8^+^ T cells

PBMCs from immunized piglets were analysed for the cellular immune response. PBMCs (1 × 10^6^ cells) were incubated with mouse anti-swine CD4^+^ and mouse anti-swine CD8^+ ^antibodies (AbD Serotec, Oxford, UK) in fluorescence-activated cell sorting (FACS) binding buffer (1% BSA, 1% serum albumin, 0.01% sodium azide) at 4 °C for 30 min. Then, the cells were washed twice with FACS binding buffer and incubated with PE-labelled mouse anti-pig CD4^+^ and FITC-labelled mouse anti-pig CD8^+^ (BD Biosciences, Franklin Lakes, NJ, USA) at 4 °C for 30 min in the dark. Thereafter, the cells were washed three times with PBS. The CD4^+^ and CD8^+^ percentages were determined by flow cytometry (BD Biosciences).

### In vitro stimulation, real-time PCR, and analysis of cytokine gene expression

Cytokine mRNA expression was analysed. Freshly isolated PBMCs were added to 24-well plates (10^7^ cells/well) with or without 1.25 μg/well *P. multocida* and incubated at 37 °C and 5% CO_2_ for 3 h. Total RNA was extracted from the stimulated or control PBMCs using TRIzol reagent (Invitrogen, CA, USA) according to the manufacturer’s instructions. Then, reverse transcription (RT) was carried out using the High-Capacity cDNA Reverse Transcription Kit (Applied Biosystems, CA, USA). Real-time PCR was carried out in the SmartCycler I system (Cepheid, Caribbean Sunnyvale, CA, USA) as described previously [13]. The primers and reaction program for swine cytokine genes and the housekeeping gene GAPDH (glyceraldehyde-3-phosphate dehydrogenase) are listed in Table 2. The expression of each gene was analysed using the relative quantification method described by Pfaffl [17]. A slope was determined from the exponential phase, under the optimized real-time PCR amplification condition, of the target gene or the reference gene (GAPDH). The amplification efficiency (E) was calculated based on the slope, where E = 10 [− 1/slope]. The mRNA expression of each target gene was calibrated by that of GAPDH at each time point and converted to the relative expression ratio (fold of induction), where fold of induction = [(E _target_) × (control CP _target_ − treatment CP _target_)]/[(E_ref_) × (control CP_ref_ − treatment CP _ref_)].

**Table 2:** **Sequences of primers for swine cytokines and GAPDH for real-time PCR**

Gene	Primer	Sequence (5′ → 3′)	Length (bp)	Accession No.^a^	Denaturation	Amplification conditions^b^ Annealing temperature
GADPH	F	TGAATTTGGCTACAGCAACAGG	186	XM_039874164.1	95 °C (5 min)	53.2 ℃ (20 s),
	R	GGTCTGGGATGGAAACTGGA				
IFN-γ	F	ACTTGGTGTTATGGTGACTG	197	X53085		57 ℃ (20 s)
	R	TAGGATGTCTAGTAGTGAG				
IL-4	F	TGACGGACGTCTTTGCTGC	178	X68330		50.8 ℃ (20 s)
	R	TCTGTGCATGAAGCCAAGAA				

F: forwards, R: reverse.

^a^GenBank accession number of cDNA and corresponding gene, available at National Center for Biotechnology Information.

^b^Denaturation at 95 ℃ for 45 s and extension at 72 ℃ for 30 s, for a total of 44 cycles.

### Statistical analysis

All data were analysed using the general linear model in the statistical software SAS (Version 9.0, Cary, NC, USA). Data are expressed as the mean ± standard deviation (SD) and were compared to the mean of the control at each time point by analysis of variance (ANOVA) and Duncan’s multiple comparisons (^a, b, c^ indicate a difference between groups). A* p* value < 0.05 was considered statistically significant.

## Results

### Expression and identification of rPMT-NC and rSly protein

The expression of rPMT-NC (98 kDa) and rSly (77 kDa) was confirmed by SDS‒PAGE and Western blotting analysis using a 1:3000 dilution of mouse anti-His or serum from mice that survived experimental challenges with live *P. multocida* or *S. suis*, respectively [11].

### Plasmid CpG can induce an IgG antibody response and protect against *P. multocida* serotype A challenge

**Figure 1:**
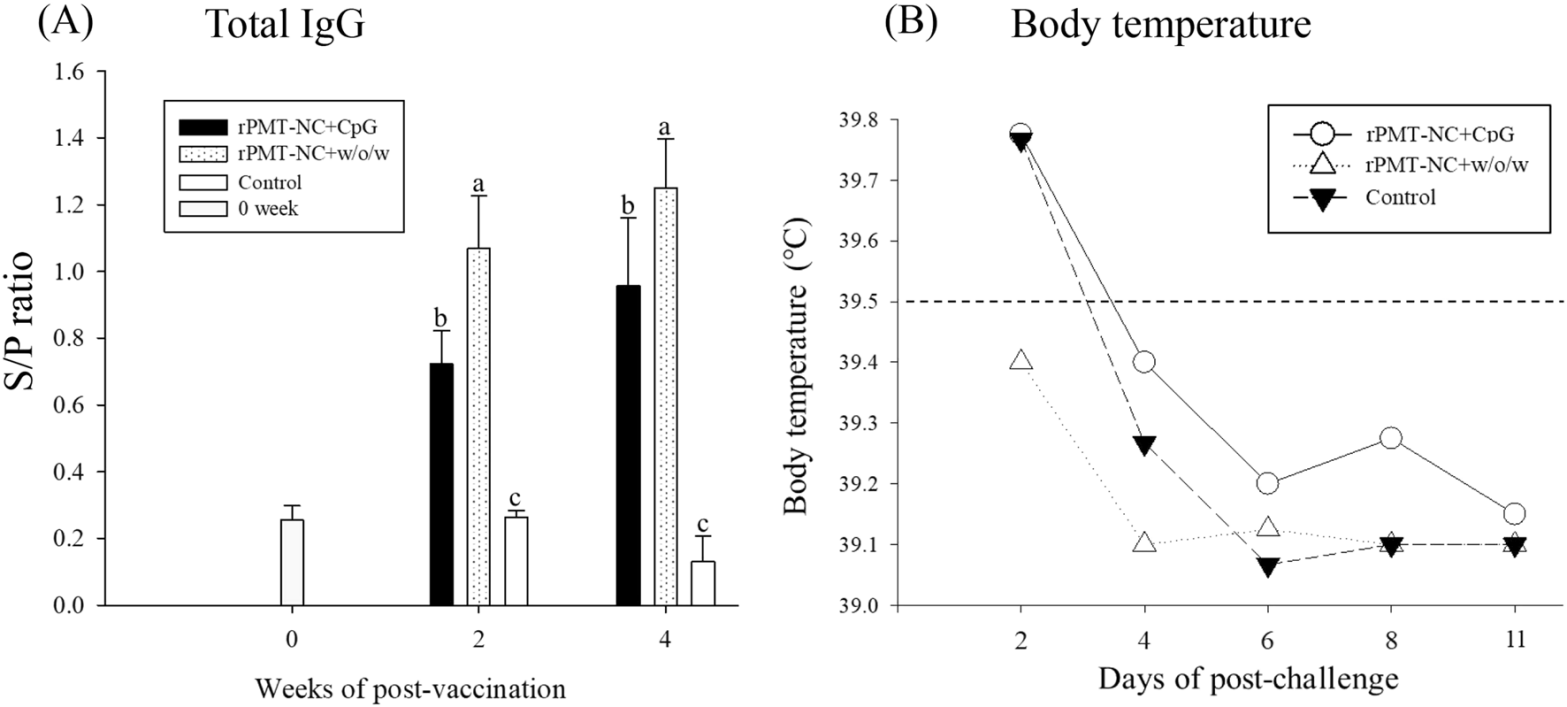
**The antibody response to** ***P. multocida* and body temperature in piglets after challenge.** Piglets were immunized twice (0 and 2 weeks) i.m. and sera were collected at 0, 2, and 4 weeks; piglets were then challenged at 4 weeks. **A** Indirect ELISA was performed with 1.25 μg/well of *P. multocida*. **B** Body temperatures recorded at 0, 2, 4, 6, 8 and 11 days are shown. S/P ratio = (Sample OD value − Negative OD value)/(Positive OD value − Negative OD value). Data are presented as the mean ± SD. Different superscript letters ^(a, b, c)^ indicate significant differences (*p* < 0.05) between treatment groups at the same time point based on Duncan’s test.

Blood samples were collected at 0, 2 and 4 weeks after immunization. The results indicated that group (1) rPMT-NC + CpG and group (2) rPMT-NC + w/o/w had significantly (*p* < 0.05) higher antibody titres than group (3) control (Figure 1A).

**Figure 2:**
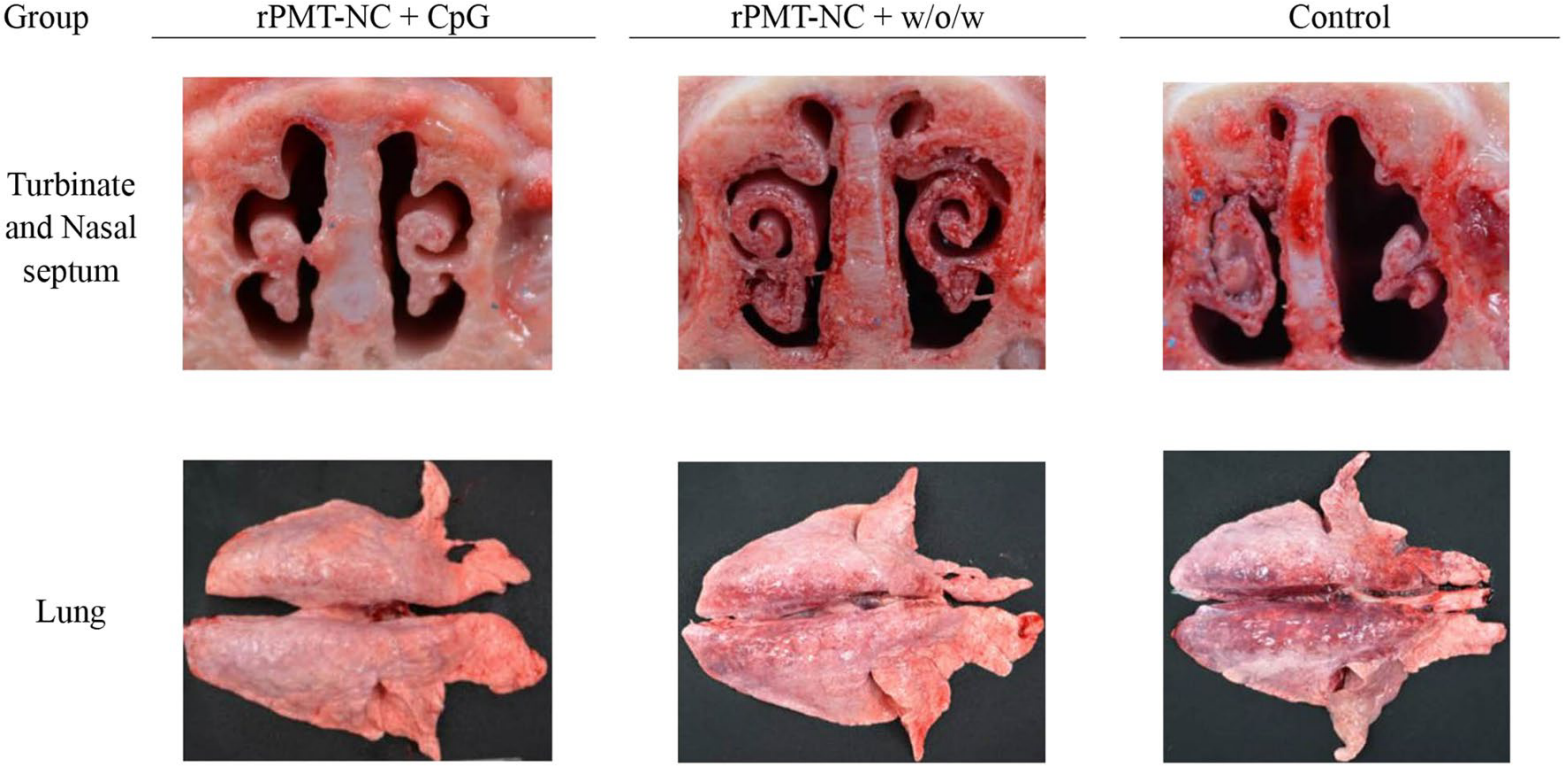
**Gross turbinate, nasal septum, and lung lesion of piglets 14 days after *P. multocida* challenge.** Piglets were immunized i.m. at week 0 and boosted at 2 weeks. All piglets were challenged intranasally with 1 × 10^8^ CFU/mL *P. multocida* serotype A 4 weeks after primary immunization. Dissection of unvaccinated piglets is shown for comparison. Characteristic gross lesions of *P. multocida* infection, including turbinate atrophy, nasal distortion and pulmonary lesions that exhibited extensive interstitial pneumonia, were observed.

All piglets were intranasally challenged with live *P. multocida* serotype A 4 weeks after primary immunization, and clinical signs were recorded. One of four piglets in the control group was seriously sick after 2 days of challenge and was sacrificed, so their data were excluded from the score. After challenge for 2 days, the CpG and control groups showed higher body temperatures than the w/o/w group (Figure 1B). The average daily weight gain of piglets in the control group (2.6 ± 2.2 kg) was less than that of the vaccinated groups (3.3 ± 1.2 and 3.8 ± 0.7 kg) (Table 3). Pathological gross lesions of turbinate atrophy and nasal distortion indicated that the control group showed more damage than the vaccinated groups (Figure 2). Moreover, the control group exhibited extensive interstitial pneumonia, while the vaccinated piglets had slight lesions (Figure 2). Pathological examination scores showed that the vaccination groups had lower clinical scores than the control group (Table 3). The recombinant protein combined with CpG or w/o/w adjuvant provided significant protection against live *P. multocida* serotype A challenge.

**Table 3:** **Clinical and pathological analysis** **of pigs after challenge with** ***P. multocida* serotype** **A**

Group	Total score^†^	The score of gross lesions			Body weight (kg)		
		Turbinate	Nasal septum	Lung	Total gain/11 days	Before challenge	After challenge
CpG (4)	15^ab^	6	3	6	3.3 ± 1.2^a^	19.1 ± 2.0	22.4 ± 1.6
w/o/w (4)	12^a^	5	2	5	3.8 ± 0.7^a^	17.9 ± 0.6	21.7 ± 0.7
Control (3)^*^	19^b^	6	4	9	2.6 ± 2.2^a^	16.0 ± 2.8	18.6 ± 2.4

^*^One of four pigs in the control group was sacrificed due to serious illness 2 days post challenge, so it was not included in the score.

^†^Differences between groups compared to the controls using Duncan’s multiple comparison. A* p* value of < 0.05 was considered significant.

### Low-dose rSly adjuvant induced antibody and T-cell responses

**Figure 3:**
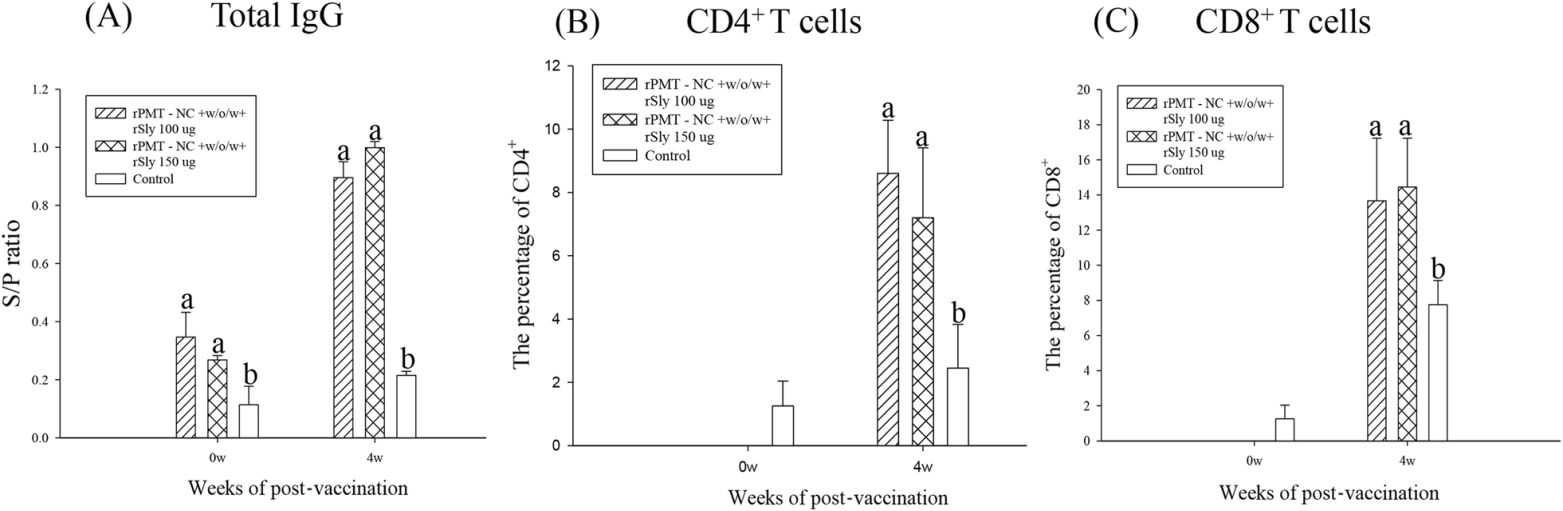
**Antibody and T-cell evaluation of the rSly adjuvant dose-dependent test.** **A** *P. multocida-*specific antibodies in sera and the percentage of **B** CD4^+^ and **C** CD8^+^ T cells in PBMCs in the rSly adjuvant dose-dependent test. Piglets were intramuscularly immunized at week 0, and sera were collected at week 0 and 4 weeks. Total IgG S/P ratio = (Sample OD value − Negative OD value)/(Positive OD value − Negative OD value). Data are presented as the mean ± SD. Different superscript letters ^(a, b, c)^ indicate significant differences (*p* < 0.05) between treatment groups at the same time point based on Duncan’s test.

Blood samples were collected at 0 and 4 weeks after immunization. Both doses induced the production of antigen-specific IgG 4 weeks after the primary vaccination, and there were no differences between the rPMT-NC + rSly (100 μg/mL) and rPMT-NC + rSly (150 μg/mL) groups (Figure 3A). In cellular responses, the percentages of CD4^+^ (8.6 ± 1.6, 7.2 ± 2.2) and CD8^+^ (13.6 ± 3.5, 14.4 ± 2.7) T cells were significantly (*p* < 0.05) increased in the vaccinated groups compared to the control group (Figures 3B and C). However, the two vaccinated groups were not different. Therefore, we selected the lower dose of rSly (100 μg/mL) as the adjuvant in our vaccine formulation.

### Biological adjuvant enhanced the antibody response and IFN-γ gene expression

**Figure 4:**
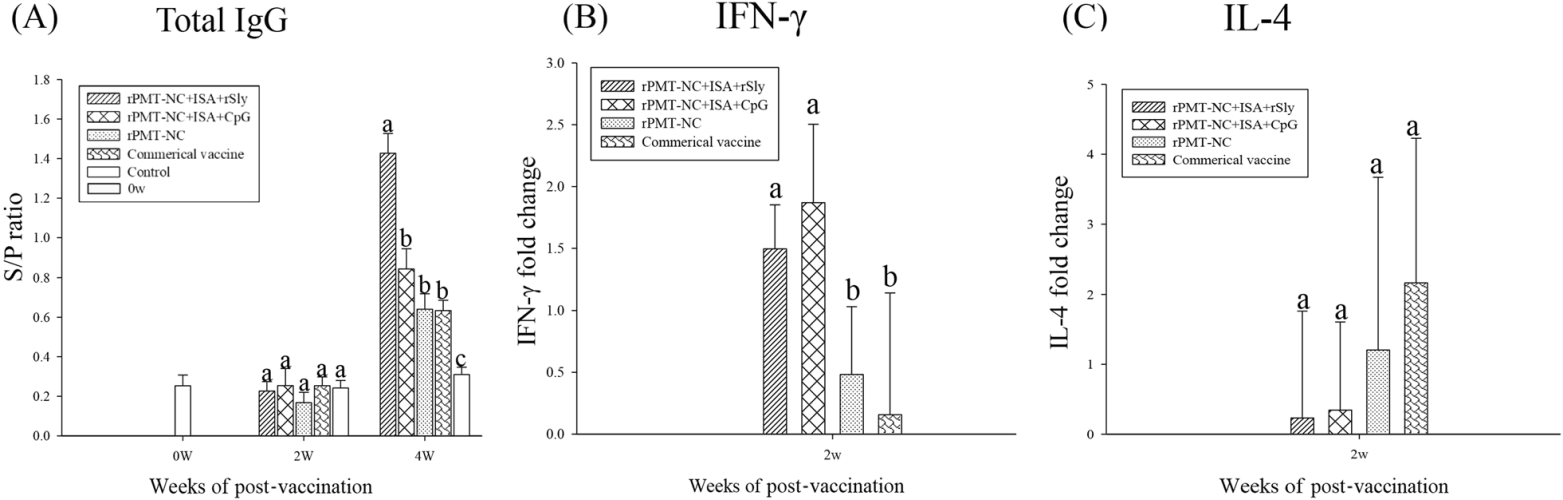
**Antibody responses and cytokine gene expression in comparison with various adjuvants.** Analysis of the titre of total IgG (**A**), cytokine IFN-γ (**B**) and IL-4 (**C**) gene expression. Piglets were intramuscularly immunized twice (week 0 and 2 weeks). Sera were collected at week 0, 2 weeks and 4 weeks, and whole blood was collected at 2 weeks after immunization. S/P ratio = (Sample OD value − Negative OD value)/(Positive OD value − Negative OD value). Data are presented as the mean ± SD. Different superscript letters ^(a, b, c)^ indicate significant differences (*p* < 0.05) between treatment groups at the same time point based on Duncan’s test.

A total of twenty 4-week-old pigs were randomly assigned to five groups. Blood samples were collected at 0, 2 and 4 weeks after immunization. The rPMT-NC + w/o/w + rSly group had significantly (*p* < 0.05) higher antibody titres than the other groups (Figure 4A).

PBMCs were isolated from vaccinated piglets on D14, and the mRNA expression of selected cytokines, including IFN-γ and IL-4, in response to stimulation with *P. multocida* in vitro was analysed. The results showed that both the rPMT-NC + w/o/w + rSly and rPMT-NC + w/o/w + CpG groups had significantly higher (*p* < 0.05) mRNA levels of IFN-γ (Figure 4B). However, there was no difference in the IL-4 response among all groups.

### Biological adjuvants reduced the number of gross pathological lesions after challenge

The protective potency of intramuscular immunization with various vaccine formulations was assessed for each group of piglets after being challenged and necropsied according to body weight and gross pathological scores based on the percentage of turbinate atrophy, nasal distortion, and lung lesions. No adverse reactions to the vaccines were observed. The average daily weight gain of piglets in the control group was less than that in the commercial vaccine group (Table 4), likely due to sickness after bacterial challenge. There was no difference among vaccine groups.

**Table 4:** **Clinical and pathological analysis of pigs** **after challenge** **with** ***P. multocida*** **serotype** **A**

Group	Total score^†^	The score of gross lesions				Body weight (kg)		
		Turbinate	Nasal septum	Lung		Total gain/11 days	Before challenge	After challenge
rPMT-NC + w/o/w + rSly	9^a^	1	0	8		4.4 ± 1.3^ab^	13.4 ± 3.8	17.7 ± 5.1
rPMT-NC + w/o/w + CpG	10^a^	4	0	6		4.9 ± 1.7^ab^	15.2 ± 1.6	20.1 ± 1.8
rPMT-NC	16^b^	4	0	12		4.8 ± 3.3^ab^	14.0 ± 4.6	18.8 ± 7.8
Commercial vaccine	18^b^	6	1	11		5.3 ± 1.0^a^	13.9 ± 1.8	17.2 ± 2.7
Control (PBS)	20^b^	8	3	9		3.0 ± 1.1^b^	11.6 ± 1.0	15.4 ± 1.5

^†^Differences between groups compared to the controls using Duncan’s multiple comparison. A* p* value of < 0.05 was considered significant

**Figure 5:**
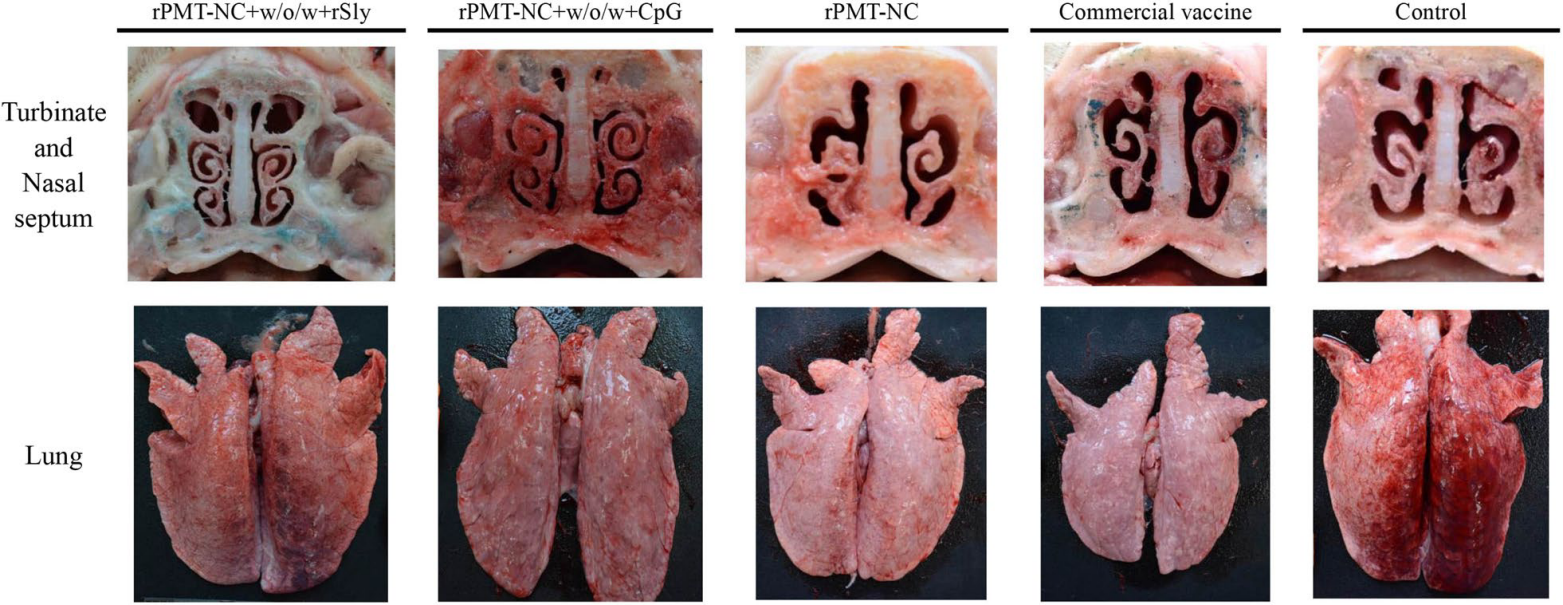
**Gross turbinate, nasal septum, and lung lesion of piglets 14 days post intranasal challenge with *P. multocida*.** Piglets were immunized i.m. at week 0 and boosted at 2 weeks. All piglets were challenged intranasally with 1 × 10^8^ CFU/mL *P. multocida* serotype A 4 weeks after primary immunization. Dissection of unvaccinated piglets is shown for comparison. Characteristic gross lesions of *P. multocida* infection, including turbinate atrophy, nasal distortion, nasal septum and pulmonary lesions that exhibited extensive interstitial pneumonia, were observed.

Pathological examination of the total score indicated that immunization with rPMT-NC protein combined with biological adjuvants (CpG, rSly) reduced turbinate atrophy, nasal distortion and lung lesions compared to the other treatments (Figure 5). The adjuvanted vaccines provided significant protection (Table 4) against live *P. multocida* serotype A challenge.

## Discussion

Vaccination is an economical and efficient way to reduce the spread of pandemic diseases affecting both humans and animals [18]. *P. multocida* is a major respiratory pathogen that causes atrophic rhinitis in pigs. A few vaccines have been developed to protect against *P. multocida* infection [19]. Traditional bacterins with toxoids are widely administered to aid protection, but several drawbacks have limited their use. Modern subunit vaccines contain several antigen fragments, have a superior safety profile to inactivated or live-attenuated vaccines, and provide cross-protection. However, the choice of antigen and the appropriate adjuvant still need to be resolved. Our previous report showed that recombinantly expressed, engineered rPMT-NC protein serves as a promising vaccine candidate in mice [11]. Furthermore, our duck vaccine study focused on identifying appropriate adjuvants showed that the constructed plasmid CpG ODN could further boost cellular immunity that is usually lacking in protein-based vaccines [6]. Hence, we sought to investigate the patented plasmid CpG as an adjuvant in piglet experiments. After immunization, the serum titre of IgG against *P. multocida* rapidly increased in the CpG and w/o/w adjuvant-containing groups (Figure 1A). Moreover, vaccinated piglets were less impacted after being challenged with live *P. multocida* serotype A, which is evidenced by clinical scores (Table 3). Our results indicate that rPMT-NC protein combined with CpG or w/o/w adjuvant could elicit protection against *P. multocida* challenge. Our current work proved that the patented plasmid CpG could also be applied in swine vaccines.

Sly is an extracellular protein of *S. suis* that can also provide cross-protection against heterologous challenges [20]. Sly triggers the activation of cytotoxic T cells, the maturation of natural killer cells, and the activation of the Th1 and Th2 immune responses [21]. A stable subunit vaccine and biological adjuvant antigen require a highly conserved region in different serotypes; therefore, Sly has been indicated as a compatible adjuvant. In our dose-dependent results, piglets administered a low dose of rSly showed an antigen-specific response based on total IgG (Figure 3A), IFN-γ gene expression and stimulated CD4^+^ and CD8^+^ T-cell populations (Figures 3B and C).

After immunization with rPMT-NC combined with rSly or plasmid CpG, the total IgG of the rSly group was significantly higher than that of the other groups (Figure 4A). After challenge with *P. multocida* serotype A, administration of vaccines that contained biological adjuvants significantly decreased gross lesions compared with the commercial or control group (Table 4 and Figure 5). CpG and rSly had similar protective effects in this study. The study findings are consistent with other research, which found that CpG combined with an oil-based adjuvant could enhance the immune response and reduce CpG concentration [22]. The results are also consistent with another study that showed that a w/o/w emulsion with CpG ODN exhibits a synergistic immunostimulatory effect [23].

In the market, most vaccines against swine atrophic rhinitis are killed bacteria (bacterin) with added segments of rPMT and formulated with Al-gel adjuvant. However, multicomponent vaccines have a complex and costly manufacturing process. Notably, when comparing our challenge data, there was no difference between the Al-gel adjuvant and rPMT without an adjuvant groups in the clinical and pathological scores after the challenge (Table 4). Al-gel is known to induce humoral immunity but is incapable of stimulating Th1-type immunity or cytotoxic T-cell responses [24]. Previous findings revealed that immunized rPMT protein with Al-gel adjuvant and toxoid groups showed similarity in offspring body weight and clinical symptoms after being challenged with the homologous strain of *P. multocida* [25].

The key benefits of our formulation are that rPMT-NC protein can serve as an antigen and rSly as an adjuvant with similar production and purification processes. These processes could be scaled up and reproduced each time to meet a more price-conscious market. As a subunit vaccine moves from the research level into the manufacturing process, cost-effectiveness must be met. In conclusion, our rSly biological adjuvant can enhance the protective efficacy of rPMT-NC against *P. multocida* challenge and has the potential to be developed as part of a cost-effective bivalent subunit vaccine against atrophic rhinitis and *S. suis* infection in pigs.

## Data Availability

The data that support the findings of this study are available from the authors upon reasonable request.
